# Supplementary material for: Saccharomyces boulardii (CNCM I-745) improves intestinal damage in sepsis by remodeling intestinal flora structure
Source: Front Cell Infect Microbiol. 2025 Jul 31;15:1602792. doi: 10.3389/fcimb.2025.1602792 (PMC12350329; doi:10.3389/fcimb.2025.1602792)
Supplement: Supplementary file 1 [file DataSheet1.zip › summary/summary/8_Advanced_analyse/2_PICRUSt2_result/KEGG/Group.DW4_vs_CW4_vs_BW4_vs_AW4/PICRUSt2_KO_Group.DW4_vs_CW4_vs_BW4_vs_AW4_diff.pdf]

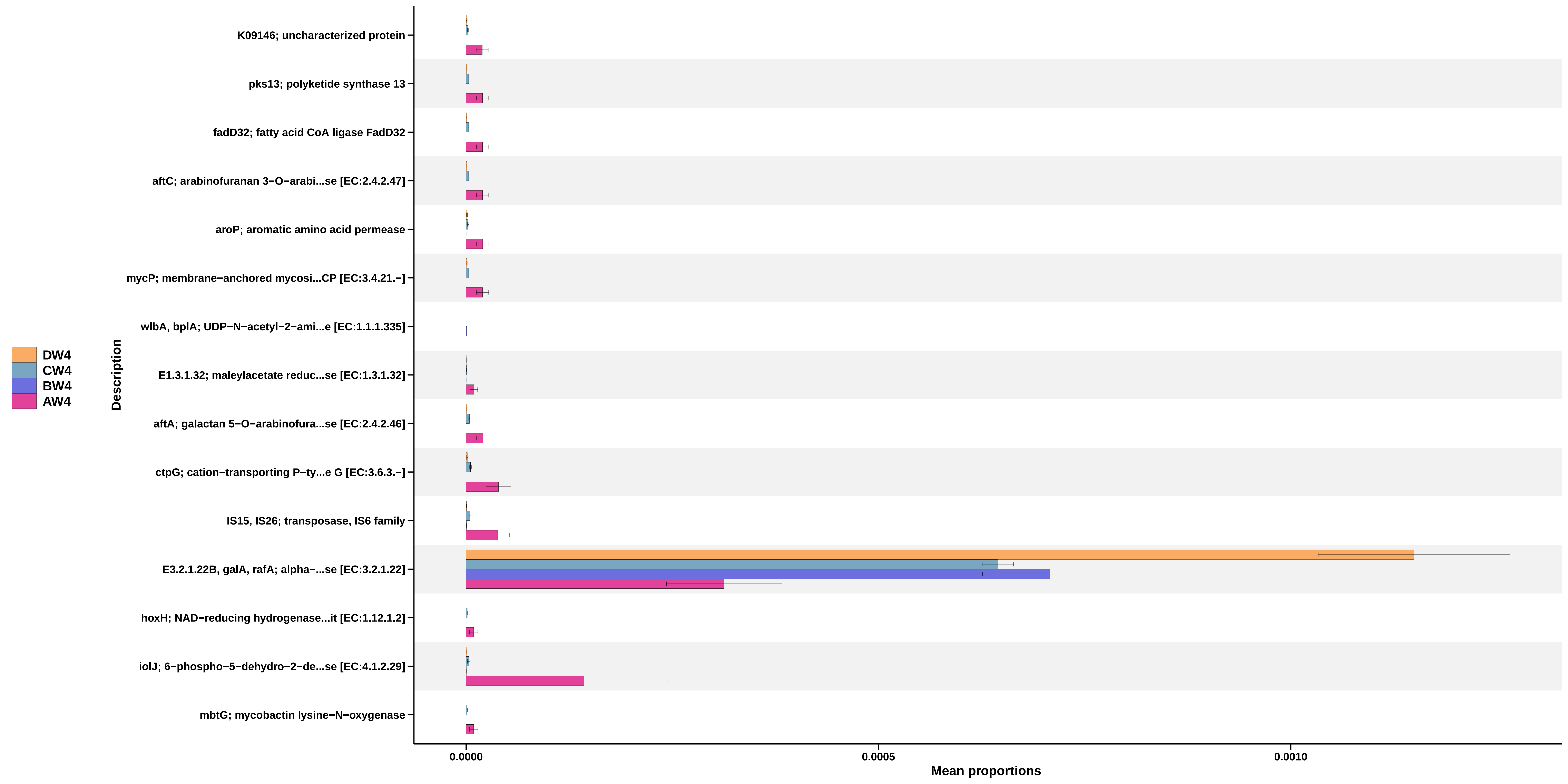

K09146; uncharacterized protein
pks13; polyketide synthase 13
fadD32; fatty acid CoA ligase FadD32
aftC; arabinofuranan 3−O−arabi...se [EC:2.4.2.47]
aroP; aromatic amino acid permease
mycP; membrane−anchored mycosi...CP [EC:3.4.21.−]
wlbA, bplA; UDP−N−acetyl−2−ami...e [EC:1.1.1.335]
E1.3.1.32; maleylacetate reduc...se [EC:1.3.1.32]
aftA; galactan 5−O−arabinofura...se [EC:2.4.2.46]
ctpG; cation−transporting P−ty...e G [EC:3.6.3.−]
IS15, IS26; transposase, IS6 family
E3.2.1.22B, galA, rafA; alpha−...se [EC:3.2.1.22]
hoxH; NAD−reducing hydrogenase...it [EC:1.12.1.2]
iolJ; 6−phospho−5−dehydro−2−de...se [EC:4.1.2.29]
mbtG; mycobactin lysine−N−oxygenase
Description
DW4
CW4
BW4
AW4
0.0000
0.0005
0.0010
Mean proportions
